# Multidisciplinary Management of Legionella Disease in Immunocompromised Patients

**DOI:** 10.7759/cureus.19214

**Published:** 2021-11-02

**Authors:** Andrew S Kao, Stephanie Myer, Madappuli Wickrama, Rana Ismail, Malitha Hettiarachchi

**Affiliations:** 1 Internal Medicine, Wayne State University School of Medicine, Detroit, USA; 2 Internal Medicine, Detroit Medical Center Sinai Grace Hospital, Detroit, USA; 3 Medicine, Detroit Medical Center Sinai Grace Hospital, Detroit, USA

**Keywords:** multidisciplinary care, immunocompromise, comorbidities, kidney transplant, legionella pneumonia

## Abstract

Legionella pneumonia is a gram-negative bacterial infection commonly associated with aerosol transmission from contaminated water sources. Impaired immunity leads to delayed clearance of infection and further predisposes individuals with Legionella pneumonia at risk of developing complications. We present a case report on a renal transplant patient with comorbid cardiac and renal dysfunction who developed community-acquired Legionella pneumonia. The case emphasizes the importance of adopting a multidisciplinary approach when managing Legionella infection in patients with multiple comorbidities and immunosuppressive states.

## Introduction

Legionnaires' disease, a severe systemic gram-negative bacterial infection caused by the Legionella pneumophila, often presents as atypical pneumonia with sub-acute fever, cough, and shortness of breath. L. pneumophila serogroup-1 accounts for almost 80% of Legionnaires' disease, and the rest accounts for the less prevalent or rare 14 serogroups [1]. The reported incidence of Legionnaires’ disease is 1.4 to 1.8 cases per 100,000 individuals in the United States, Europe, and Australia [2-4]. According to the World Health Organization, the mortality rate may reach as high as 80% in untreated immunosuppressed individuals and decline to 5%-30% with prompt and appropriate management [5]. While the etiology is associated with aerosol transmission from contaminated water sources in community-acquired and hospital-acquired settings, inhaling fine aerosolized droplets contaminated with Legionella from cooling towers, humidifiers, showers, and air conditioners increase access to lung airways and cause Legionella pneumonia [6]. Having chronic medical conditions and being immunocompromised further increase Legionella infection susceptibility, leading to a gradual decline in pulmonary function and an overall rapid clinical deterioration if left untreated. This case report describes the importance of adopting a multidisciplinary management approach when caring for an immunocompromised patient who contracted Legionella pneumonia.

## Case presentation

A 63-year-old African American male presented with a two-day history of intermittent fever and shortness of breath; he complained of productive cough without specified sputum characteristics, concomitant abdominal discomfort, lower back pain, and watery diarrhea. His medical history was significant for heart failure with reduced ejection fraction, chronic kidney disease, type 2 diabetes, and obstructive sleep apnea. Ten years ago, the patient underwent a right kidney transplant due to poorly controlled hypertension and received post-transplant tacrolimus and mycophenolate immunosuppressive therapy. He lives by himself, and upon further investigation into his home environment, he reported having two air conditioning systems at least 40 years old. On admission, he was febrile, tachycardic, tachypneic, and hypoxic. Physical exam revealed dehydration signs and decreased breath sounds on the left upper and lower lung fields with positive rhonchi and bilateral pitting edema in lower extremities.

**Figure 1:**
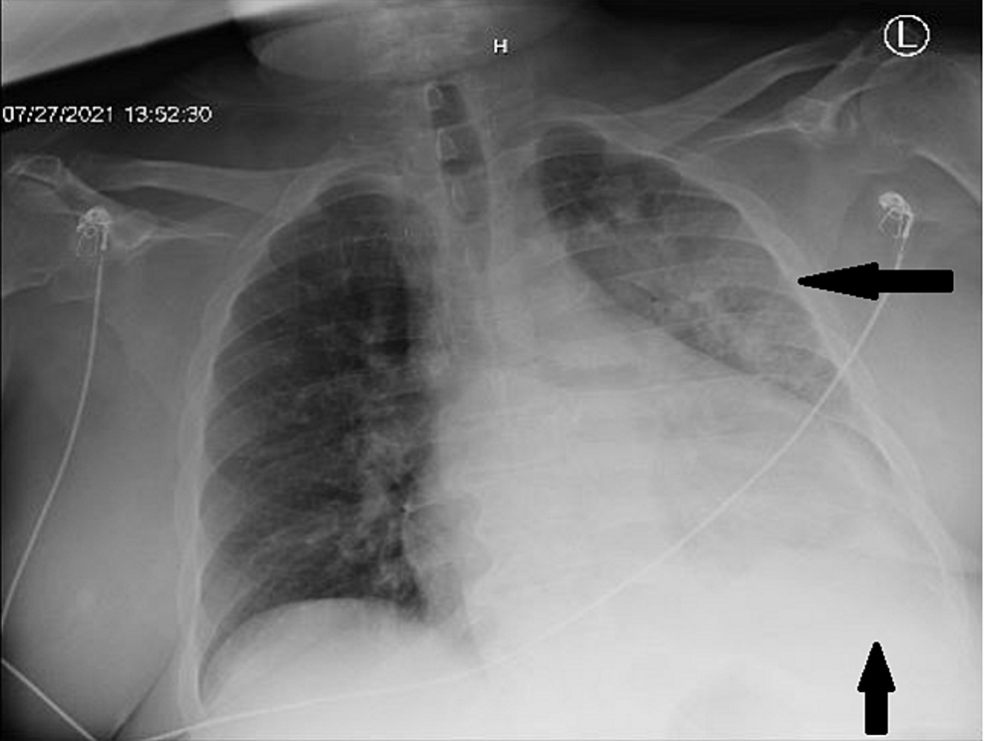
Initial presentation on chest x-ray showing asymmetric left lung area of consolidation with small left effusion.

**Figure 2:**
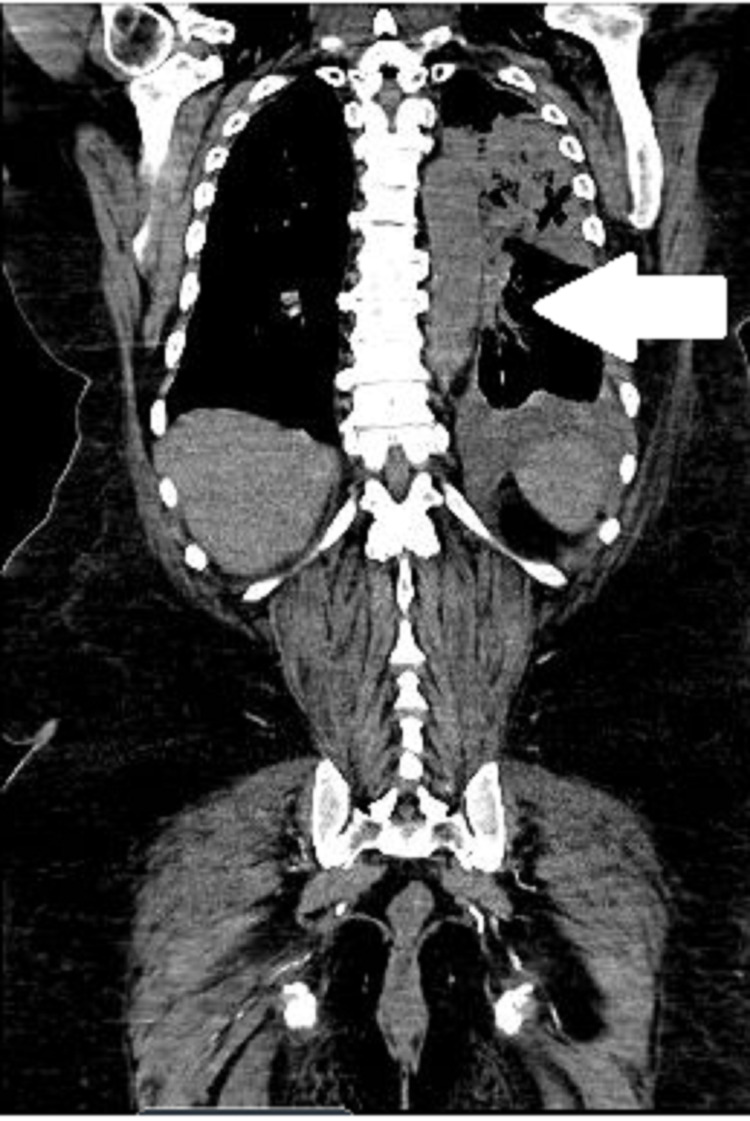
CT chest and thorax showing multiple enlarged mediastinal lymph nodes measuring up to 1.3 cm. Other left hilar and lower mediastinal lymph nodes are also noted to be enlarged.

The metabolic panel was pertinent for elevated creatinine of 2.79 mg/dL from a baseline of 2.15 mg/dL; CBC was remarkable for leukocytosis with left shift and normocytic anemia with hemoglobin at 9.5 g/dL. Urinalysis revealed +3 bacteriuria and +3 hematuria. Urine Legionella antigen was positive. EKG showed atrial fibrillation with a rapid ventricular response, multiple premature atrial and ventricular complexes with prolonged QTc at 492. Chest x-ray showed cardiomegaly in addition to left upper lobe pneumonia with mild left pleural effusion (Figure 1). Subsequent thorax/chest CT scan detected multifocal pneumonia notably concentrated in the left upper lobe with small left-sided pleural effusion, moderate cardiomegaly with trace pericardial effusion, and mediastinal, hilar adenopathy (Figure 2).

The patient was started on 0.9% NaCl fluid resuscitation and azithromycin that later changed to the fourth-generation fluoroquinolone (moxifloxacin) due to possible drug interaction with tacrolimus. The nephrology team closely monitored and daily adjusted tacrolimus. Hydration was maintained cautiously to prevent the worsening of acute kidney injury. An echocardiogram revealed a 30% reduced ejection fraction, consistent with a previous echo. Cardiology followed up during hospital stay and recommended outpatient evaluation of automatic implantable cardioverter-defibrillator (AICD) placement. He was placed on BIPAP at night and nasal cannula oxygen at daytime. Due to concerns over mobility and restrictive life space in the home environment, physical and occupational therapy recommended subacute rehabilitation (SAR) placement. The patient completed eight days of moxifloxacin and was discharged to rehabilitation.

## Discussion

L. pneumophila mainly targets the alveolar macrophages and replicates intracellularly in phagosomal vacuoles [7]. The infected macrophages release chemokines and cytokines that trigger a host cell-mediated pro-inflammatory response. When L. pneumophila replication peaks, the phagosomes merge with lysosomes, leading to lysed vacuoles and host cell destruction. More infectious agents are released into the body to cause a wider spread, primarily in the lungs [8]. According to recent cohort studies, several risk factors correlate with Legionella infection, most notably, male gender and older age starting at 50 years onward [9]. Individuals with noncommunicable diseases such as diabetes, heart disease, kidney failure, and cancer are also at higher risk [10]. Immunosuppression treatment of a solid organ transplant weakens host immunity and increases the risk of acquiring L. pneumophila [11]. For 10 years, our patient was on immunosuppressive therapy of prednisone, tacrolimus, mycophenolate and developed myelosuppression as a side effect. Although our patient's heart failure condition with reduced ejection fraction and diabetes mellitus were well-controlled, the effect of long-term immune system suppression increased his vulnerability for L. pneumophila.

The clinical management of Legionella in an immunocompromised patient requires a multidisciplinary approach. For our patient, the initial pharmacotherapy approach used to treat Legionella pneumonia conflicted with his immunosuppressive therapy. Hence, the infectious disease team focused on treating Legionella pneumonia and recommended an antibiotic regimen that minimized the drug clearance burden on the patient's transplanted kidney, while the nephrology team monitored his kidney function and adjusted tacrolimus dosage accordingly. Additionally, the cardiology team ensured that the patient's heart failure condition and atrial fibrillation were well-controlled. The primary internal medicine team served as the bridge to connect treatment goals of all individual teams and closely monitored the hospital and clinical disease course.

Among patients with medical comorbidities, first-line treatment includes the administration of fluoroquinolone or macrolides with rifampin [12,13]. In our patient, the infectious disease team recommended against the latter due to drug-drug interaction with calcineurin inhibitors (tacrolimus). Macrolides and erythromycin are potent inhibitors of cytochrome P450 isoform 3A4, which metabolizes calcineurin inhibitors such as tacrolimus [14]. Clinical cases have shown that the initiation of erythromycin twice per day in a patient on immunosuppressive therapy would induce a six-fold increase in tacrolimus concentrations after only two days of erythromycin [15]. Monotherapy with fluoroquinolones has shown to be more effective than macrolides with rifampin in treating Legionella pneumonia [16]. In our case, azithromycin initiated in the emergency department was discontinued, and moxifloxacin was started instead during the hospital course considering his history of renal transplant and chronic kidney injury.

Multiple complications may arise when caring for immunocompromised patients with Legionella pneumonia. Lung pathologies such as abscesses, cavitations, complicated pleural effusions, or empyema are commonly seen particularly in those with solid organ transplants [17]. Moreover, extrapulmonary involvement of integumentary and cardiovascular systems typically occurs in immunocompromised hosts. Cutaneous lesions such as erythema, nodules, induration, and ulcers have been reported in patients with solid organ transplants, malignancies, and prolonged use of steroids therapy [18]. Although rare, non-infectious aortitis can occur among transplant recipients and dialysis patients [19]. Overall, chronic immunosuppression in post-organ transplantation increases the host’s susceptibility to a cascade of complications and infections, thus requiring a multidisciplinary clinical approach to mitigate the complexities.

## Conclusions

Current clinical evidence shows that immunosuppression increases the risk of community-acquired Legionella pneumonia despite its rare occurrence. Kidney transplants, heart failure, and other chronic medical conditions accompanying Legionella infection predispose patients to impending mortality if not addressed promptly. The impaired immune system further worsens lung injury, leading to the extrapulmonary spread of systemic cutaneous and cardiovascular complications. In our patient, post-transplant immunosuppression required close monitoring and titration in conjunction with an antibiotic regimen to avoid organ rejection. The primary team coordinated the infectious disease and nephrology teams' recommendations, which focused on treating the active Legionella infection without compromising the existing comorbidities. This case exemplifies successful teamwork and collaboration among different specialties to provide high-quality, life-saving patient-centered care.
